# Combined exposure to polychlorinated biphenyls and high-fat diet modifies the global epitranscriptomic landscape in mouse liver

**DOI:** 10.1093/eep/dvab008

**Published:** 2021-09-17

**Authors:** Carolyn M Klinge, Kellianne M Piell, Belinda J Petri, Liqing He, Xiang Zhang, Jianmin Pan, Shesh N Rai, Kalina Andreeva, Eric C Rouchka, Banrida Wahlang, Juliane I Beier, Matthew C Cave

**Affiliations:** Department of Biochemistry and Molecular Genetics, University of Louisville School of Medicine, Louisville, KY 40292, USA; University of Louisville Center for Integrative Environmental Health Sciences (CIEHS), Louisville, KY 40292, USA; Department of Biochemistry and Molecular Genetics, University of Louisville School of Medicine, Louisville, KY 40292, USA; Department of Biochemistry and Molecular Genetics, University of Louisville School of Medicine, Louisville, KY 40292, USA; Department of Chemistry, University of Louisville College of Arts and Sciences, Louisville, KY 40292, USA; Department of Chemistry, University of Louisville College of Arts and Sciences, Louisville, KY 40292, USA; University of Louisville Hepatobiology and Toxicology Center, Louisville, KY 40292, USA; University of Louisville Alcohol Research Center, Louisville, KY 40292, USA; University of Louisville Center for Integrative Environmental Health Sciences (CIEHS), Louisville, KY 40292, USA; Biostatistics and Bioinformatics Facility, James Graham Brown Cancer Center, University of Louisville School of Medicine, Louisville, KY 40292, USA; University of Louisville Center for Integrative Environmental Health Sciences (CIEHS), Louisville, KY 40292, USA; University of Louisville Hepatobiology and Toxicology Center, Louisville, KY 40292, USA; University of Louisville Alcohol Research Center, Louisville, KY 40292, USA; Department of Bioinformatics and Biostatistics, University of Louisville School of Public Health and Information Sciences, Louisville, KY 40292, USA; Biostatistics and Bioinformatics Facility, James Graham Brown Cancer Center, University of Louisville School of Medicine, Louisville, KY 40292, USA; The University of Louisville Superfund Research Center, Louisville, KY 40292, USA; Bioinformatics and Biomedical Computing Laboratory, Department of Computer Engineering and Computer Science, JB Speed School of Engineering, University of Louisville, Louisville, KY 40292, USA; Department of Biochemistry and Molecular Genetics, University of Louisville School of Medicine, Louisville, KY 40292, USA; The University of Louisville Superfund Research Center, Louisville, KY 40292, USA; Division of Gastroenterology, Hepatology & Nutrition, Department of Medicine, University of Louisville School of Medicine, Louisville, KY 40292, USA; Department of Medicine, Division of Gastroenterology, Hepatology & Nutrition, University of Pittsburgh, Louisville, KY 40292, USA; Pittsburgh Liver Research Center (PLRC), Louisville, KY 40292, USA; Department of Environmental and Occupational Health Pittsburgh, University of Pittsburgh, Pittsburgh, PA 15213, USA; Department of Biochemistry and Molecular Genetics, University of Louisville School of Medicine, Louisville, KY 40292, USA; University of Louisville Center for Integrative Environmental Health Sciences (CIEHS), Louisville, KY 40292, USA; University of Louisville Hepatobiology and Toxicology Center, Louisville, KY 40292, USA; University of Louisville Alcohol Research Center, Louisville, KY 40292, USA; The University of Louisville Superfund Research Center, Louisville, KY 40292, USA; Division of Gastroenterology, Hepatology & Nutrition, Department of Medicine, University of Louisville School of Medicine, Louisville, KY 40292, USA; Department of Pharmacology and Toxicology, University of Louisville School of Medicine, Louisville, KY 40292, USA

**Keywords:** epitranscriptomics, PCBs, high-fat diet, liver, transcriptomics, writers, readers

## Abstract

Exposure to a single dose of polychlorinated biphenyls (PCBs) and a 12-week high-fat diet (HFD) results in nonalcoholic steatohepatitis (NASH) in mice by altering intracellular signaling and inhibiting epidermal growth factor receptor signaling. Post-transcriptional chemical modification (PTM) of RNA regulates biological processes, but the contribution of epitranscriptomics to PCB-induced steatosis remains unknown. This study tested the hypothesis that PCB and HFD exposure alters the global RNA epitranscriptome in male mouse liver. C57BL/6J male mice were fed a HFD for 12 weeks and exposed to a single dose of Aroclor 1260 (20 mg/kg), PCB 126 (20 µg/kg), both Aroclor 1260 and PCB 126 or vehicle control after 2 weeks on HFD. Chemical RNA modifications were identified at the nucleoside level by liquid chromatography-mass spectrometry. From 22 PTM global RNA modifications, we identified 10 significant changes in RNA modifications in liver with HFD and PCB 126 exposure. Only two modifications were significantly different from HFD control liver in all three PCB exposure groups: 2ʹ-O-methyladenosine (Am) and N(6)-methyladenosine (m6A). Exposure to HFD + PCB 126 + Aroclor 1260 increased the abundance of N(6), O(2)-dimethyladenosine (m6Am), which is associated with the largest number of transcript changes. Increased m6Am and pseudouridine were associated with increased protein expression of the writers of these modifications: Phosphorylated CTD Interacting Factor 1 (PCIF1) and Pseudouridine Synthase 10 (PUS10), respectively, in HFD + PCB 126- + Aroclor 1260-exposed mouse liver. Increased N1-methyladenosine (m1A) and m6A were associated with increased transcript levels of the readers of these modifications: YTH N6-Methyladenosine RNA Binding Protein 2 (YTHDF2), YTH Domain Containing 2 (YTHDC2), and reader FMRP Translational Regulator 1 (FMR1) transcript and protein abundance. The results demonstrate that PCB exposure alters the global epitranscriptome in a mouse model of NASH; however, the mechanism for these changes requires further investigation.

## Introduction

Chronic environmental exposures to ubiquitous synthetic chemicals including polychlorinated biphenyls (PCBs) contribute to metabolic diseases including toxicant-associated steatohepatitis (TASH), a form of nonalcoholic fatty liver disease (NAFLD) [1, 2]. Fatty liver disease may progress from steatosis to steatohepatitis [nonalcoholic steatohepatitis (NASH), featuring increased liver injury and inflammation with or without fibrosis], to cirrhosis and hepatocellular carcinoma (HCC) [3]. The annual mortality from cirrhosis has increased by 65%, and the annual death rate from liver cancer has doubled since 1999 [4]. PCBs are positively associated with TASH, HCC, aberrant liver enzyme profiles and mortality in human cohorts (reviewed in [2]). PCBs are endocrine- and metabolism-disrupting chemicals that promote obesity, type 2 diabetes and/or fatty liver in humans and animals (reviewed in [2]). PCB exposures are considered the first ‘hit’ promoting steatohepatitis due to the second ‘hit’ of nutrient stress from a high-fat diet (HFD) [2].

We demonstrated that a single exposure of male C57Bl/6J mice to Aroclor 1260 [a non-dioxin-like (NDL) PCB mixture] altered the hepatic proteome, including increased macrophage infiltration detected as increased Cd68 transcript levels, and attenuated the liver’s protective responses against chronic (12 weeks) HFD nutritional stress [5]. Interestingly, we recently demonstrated that a single exposure to the dioxin-like (DL) PCB 126 produced a distinct hepatic proteome in male C57Bl/6J mice compared to Aroclor 1260, including the suppression of pro-fibrotic gene expression [6]. The PCB 126-exposed mice also showed a decreased plasma alanine aminotransferase (ALT), suggesting a protective effect of PCB 126 exposure from HFD-induced liver stress. Exposure to a single combined dose of both Aroclor 1260 and PCB 126 was associated with Gene Ontology (GO) processes related to epigenetic mechanisms [6].

Over 160 chemical modifications of transcribed RNAs that regulate transcript fate and function have been identified [7]. The proteins that add the chemical modification, recognize the specific modified RNA and remove that modification are termed ‘writers, readers, and erasers’. Transfer RNAs (tRNAs) are the most extensively modified cellular RNAs with 11–13 modifications per tRNA [8]. Many chemical modifications are found uniquely in tRNA within the D, T and anticodon loops that regulate tRNA stability, folding and interaction with the Elongator complex for translational fidelity [9]. One hundred and thirty modifications have been identified in ribosomal RNA (rRNA), including isomerization of uridine to pseudouridine (Ψ) and 2′-O-methylation of the ribose (Am) [8]. There are 13 known internal messenger RNA (mRNA) modifications located in the 5ʹ and 3ʹ untranslated regions (UTRs) as well as exons and introns [10]. N(6)-methyladenosine (m6A) is the most common dynamic modification of the transcriptome and regulates the function and processing of mRNAs, long non-coding RNAs (lncRNAs) and primary microRNAs (pri-miRNAs) [11]. In mammalian mRNA, ∼0.1–0.4% of RNA are exposed to m6A modification, with an average of 3–5 m6A sites per transcript [12]. The mechanisms regulating and roles of epitranscriptomic RNA modifications other than m6A are largely unknown [13].

Few studies have examined the impact of environmental chemicals on the epitranscriptome, the vast majority focused on m6A, and only in a small number of cells or tissues (reviewed in [14]). Likewise, readers, writers and erasers regulating N1-methyladenosine (m1A) and 5-methylcytidine (m5C) have been examined at the transcript level in cell lines and zebrafish. For mouse liver, only the impact of copper exposure has been examined. None of these studies examined levels of m6A, m1A or m5C *in vivo*, and the authors of this review noted the challenge of measuring RNA modifications represents a significant barrier in the field of environmental toxicology [14]. Thus, the overall objective of this study was to identify the impact of NDL versus DL PCBs alone or in combination with the global RNA epitranscriptome in the liver of HFD-fed male mice and to evaluate the contribution of these chemical RNA modifications on the PCB-specific changes in the proteome [6] and transcriptome.

Here, we tested the hypothesis that a single PCB exposure alters the global RNA epitranscriptome in HFD-fed male mouse liver. We identified 10 significant changes in global post-transcriptional chemical modification (PTM) RNA modifications in C57Bl/6J male mouse liver RNA after a 12-week *in vivo* exposure to HFD after a single dose of Aroclor 1260 and PCB 126 or combined Aroclor 1260 + PCB 126 using mass spectrometry (MS) since the resolution of MS allows identification of low-abundance chemical modifications [15]. We examined the readers, writers and erasers of these epitranscriptomic marks in the proteome of these liver samples [6] and in a new RNA-sequencing (RNA-seq) transcriptome analysis of the same liver samples. Using a comprehensive bioinformatics approach, we identified networks associated with the epitranscriptome and examined the expression of hub proteins and transcripts in these networks.

## Materials and Methods

### Animal Studies

**Figure 1:**
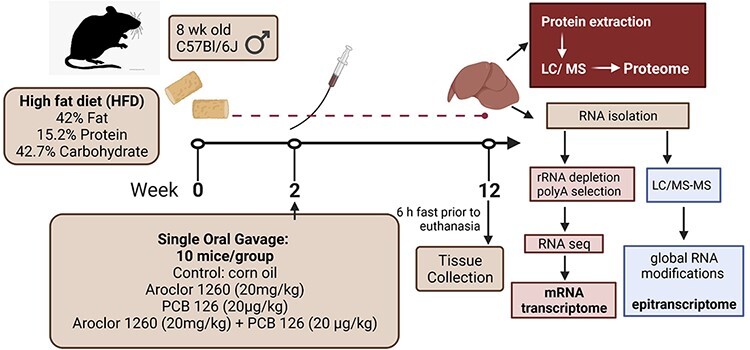
Experimental design: 8-week-old male C57Bl6/J mice were randomized into four groups of 10 mice/group. Mice received HFD diet (TekLad TD88137) for 2 weeks prior to oral gavage of corn oil vehicle (control), Aroclor 1260 (20 mg/kg NDL-PCB), PCB 126 (20 µg/kg) or the combination of Aroclor 1260 (20 mg/kg) + PCB 126 (20 µg/kg). After 12 weeks, the livers were collected and proteins extracted for proteome analysis [6]. RNA was isolated from five livers/group using Trizol. RNA was processed for RNA-seq to identify the mRNA transcriptome or for LC–MS/MS analysis of chemical RNA modifications in the global epitranscriptome. Created with BioRender.com

The experimental design is modeled in Fig. 1. The animal protocol was ratified by the University of Louisville Institutional Animal Care and Use Committee [6]. Adult male C57BL/6 mice (8 weeks old) were purchased from Jackson Laboratory and randomized into four equal groups (*n* = 10). All mice were fed *ad libitum* a HFD (15.2%, 42.7% and 42.0% of total calories from protein, carbohydrate and fat, respectively; TekLad TD88137) throughout the study. At 10 weeks of age, 10 mice in each group were given either corn oil, Aroclor 1260 (20 mg/kg), PCB 126 (20 μg/kg) or a mixture of Aroclor 1260 (20 mg/kg) plus PCB 126 (20 μg/kg) via a one-time oral gavage. These concentrations were selected based on previous studies showing that Aroclor 1260 and PCB 126 act as ‘second hits’ in HFD-fed mice to induce steatohepatitis in a chronic mouse exposure model [16]. While Aroclor 1260 contains the PCB congeners that are reflective of human adipose bioaccumulation patterns [17], it did not activate mouse Ahr at environmentally relevant doses [18]. Because humans are exposed simultaneously to DL and NDL PCB mixtures, Aroclor 1260 alone does not adequately represent human exposure to PCB mixtures. Therefore, PCB 126 was added to Aroclor 1260. The dose of Aroclor 1260 in this study is the same as in our previous studies [16, 18]. Because PCB 126 is not the only DL PCB congener and human exposures vary, the PCB 126 dose was increased to 0.1% (20 μg/kg) [16]. We note that the 20-μg/kg PCB 126 dose is much lower than the 1.6-g/kg dose used in other studies [19]; however, the 20-μg/kg PCB 126 dose was sufficient to increase *Cyp1a2* transcript expression, as an example of an AhR target gene [18]. On the other hand, Aroclor 1260 (20 mg/kg) did not induce *Cyp1a2*, most likely due to the low levels of DL PCBs in the Aroclor 1260 mixture, which were not sufficient to activate the AhR [16]. The total time of HFD exposure was 12 weeks, with 10 weeks of co-PCB exposure after the single oral gavage (Fig. 1). At the end of week 12, post-gavage, the mice were fasted for  ∼6 h prior to euthanasia, and liver samples were harvested as described [6].

### RNA Isolation and Digestion

RNA was isolated from frozen mouse livers (stored at −80°C) [6]. RNA was isolated from five mouse livers/exposure group using Trizol (ThermoFisher) according to the manufacturer’s instructions. RNA quality and concentration were evaluated using an Agilent 2100 Bioanalyzer (Agilent Technologies, Santa Clara, CA). Total RNA was denatured at 90°C for 3 min and then chilled in ice water for 3 min. To digest RNAs, 15 µg RNA was first mixed with 240 units nuclease S1 (Promega, Madison, WI) and 2 μl of reaction buffer (500 mM sodium acetate, 2.8 M NaCl and 45 mM ZnSO_4_, pH = 4.5). The mixture was incubated at 37°C for 4 h. After removing nuclease S1 using a Microcon centrifugal filter (Microcon YM-10, Sigma-Aldrich, St. Louis, MO), the pH of the solution was adjusted to basic using 2.5 µl of a mixture (1 mM MgCl_2_, 0.1 mM ZnCl_2_, 1 mM spermidine, 50 mM Tris-HCl, pH = 9.3), and 0.5 units of phosphodiesterase I (Worthington Biochemical Corporation, Lakewood, NJ) was added. The solution was then incubated at 37°C overnight. Phosphodiesterase I was removed using a Microcon YM-10 filter. Finally, 3 units of antarctic phosphatase (Promega, Madison, WI) was added, and the solution was incubated at 37°C for 4 h. Antarctic phosphatase was removed using a Microcon YM-3 filter (Sigma-Aldrich, St. Louis, MO).

### LC–MS/MS Analysis

All samples were analyzed in random order on a Thermo Q Exactive HF Hybrid Quadrupole-Orbitrap Mass Spectrometer coupled with a Thermo DIONEX UltiMate 3000 HPLC system (Thermo Fisher Scientific, Waltham, MA, USA). The UltiMate 3000 HPLC system was equipped with a reversed-phase chromatography column [20]. To obtain full MS data, every sample was analyzed by liquid chromatography (LC)–MS in positive mode (+). For metabolite identification, one pooled sample for each group was analyzed by LC–MS/MS in positive mode to acquire MS/MS spectra at three collision energies (20, 40 and 60 eV).

### Data Processing

XCMS software was used to deconvolute LC–MS spectra [21]. MetSign software was used for cross-sample peak list alignment, normalization and statistical analysis [22, 23]. To identify nucleosides, the LC–MS/MS data of the pooled samples were matched to the MS/MS spectra of 94 nucleoside standards recorded in Compound Discoverer software 3.2 MzVault (Thermo Scientific) that contains parent ion *m*/*z* value, retention time and MS/MS spectra. Chemspider and mzCloud were used for the identification of unknown compounds [24]. The threshold for the spectral similarity of the MS/MS spectra of a nucleoside standard and a spectrum of the pooled sample was set as  ≥40 with a maximum score of 100, while the thresholds of the retention time difference and *m*/*z* variation window were set as  ≤0.15 min and  ≤5 ppm, respectively.

### Statistical Analysis

SATP was used to analyze the differential expression analysis for each identified chemical RNA modification [25, 26]. The quantile method was used to normalize the intensity data after logarithm transformation with base 2. Then, we used LIMMA/Moderated t-test to compare the vehicle control to Aroclor 1260, PCB 126 or both Aroclor 1260 and PCB 126. For each comparison, the logarithm of fold change, *P*-value and adjusted *P*-value using the Benjamini–Hochberg (BH or FDR) [27] method for multiple testing of chemicals were evaluated.

### RNA-Sequencing

RNA was isolated from five mouse livers/experimental exposure using the RNeasy Mini Kit (Qiagen, Gaithersburgh, MD) according to manufacturers’ protocol. RNA-seq was performed as previously described [28, 29] with minor modifications. Briefly, libraries were prepared from 1 µg of mouse liver RNA using the TruSeq stranded mRNA Library Prep Kit (Illumina, San Diego, CA), along with TruSeq RNA CD Index Plate. Following preparation, the library was validated for size, purity and semiquantitation on an Agilent Bioanalyzer (Agilent Technologies, Santa Clara, CA). Sequencing library quantitation was done by performing a MiSeq Nano (Illumina) test run. Samples were denatured and diluted to a final concentration of 1.8 pM prior to loading into the cartridge. Two sequencing runs were performed on the Illumina NextSeq 500 using the NextSeq 500/550 High Output Kit v2.5 (75 cycles). Data as fastq files were downloaded from Illumina’s BaseSpace onto the KBRIN server for analysis. Quality control of the raw sequence data was performed using FastQC (version 0.10.1) for each sequencing run, and the sequences were considered to be high quality, and no sequence trimming was necessary. The sequences were aligned to the mouse reference genome assembly (GRCm38.p6) using STAR (version 2.6) [30]. Differential expression was performed using DESeaq2 [31]. The raw counts were obtained from the STAR aligned bam format files using HTSeq (version 0.10.0) [32]. The raw counts were normalized using the relative log expression method and then filtered to exclude genes with <10 counts across samples. The counts were then filtered to include only genes with minimum expression of 1 FPKM in three or more samples and an average of at least 1 FPKM. Entrez gene identifiers for significantly expressed genes had a *q*-value cutoff of 0.05. The raw data of the RNA-seq are available at the Gene Expression Omnibus database (accession number GSE173271).

### *In Silico* Network Analysis

Data from RNA-seq were analyzed such that transcripts selected had a log2 fold-change (log2FC) >0.34 (or −0.34 for repressed transcripts) and a statistically significant threshold *q* value <0.05. Network analysis for RNA modifications, the DEG in the mRNA transcriptome and the proteome from these liver samples [6] was evaluated using the web-based software MetaCore version 21.1 (Cortellis, Philadelphia, PA). MetaCore is a manually curated database of experimental findings and interactions, including protein–protein, protein–DNA, protein–RNA and protein–compounds; metabolic and signaling pathways; and other additional information.

## Results

### Identification of Chemical Modifications in Total Mouse Liver RNA

PCBs, both NDL and DL congeners, are metabolism-disrupting chemicals that result in obesity-related diseases including NAFLD in exposed human and animal populations (reviewed in [2]). We previously reported large differences (99.58%) in the liver proteome of male C57Bl6/J mice exposed to HFD and either the NDL Aroclor 1260 or DL PCB congener PCB 126 alone or in combination 12 weeks after a single oral gavage of vehicle control (corn oil) or the PCB [6] (study design in Fig. 1). To examine if post-transcriptional RNA modifications are associated with the combined PCB and HFD exposure and may contribute to these proteome differences, LC–MS/MS analysis of total RNA isolated from mouse liver was used to identify and quantify the abundance of the modified and unmodified nucleosides [20]. Twenty-two PTMs of RNA were detected in the mouse liver samples (Supplementary Table S1), and 10 RNA modifications were statistically significant compared to HFD control in at least one PCB exposure group (Table 1). The writers, erasers, readers and function of these 10 significant RNA modifications are summarized in Table 2.

**Table 1:** PCB exposures alter chemical modifications identified in total RNA from male C57Bl/J mouse liver with HFD (Values are the mean logFC from five independent mice/exposure group, where logFC is the logarithm of the fold change with base 2 and the adjusted (Adj.) *P* value is the adjustment for the raw *P*-value using BH method for multiple testing of chemicals [27].)

RNA modification	PubChem CID	Aroclor 1260 logFC	Aroclor 1260 Adj. *P*-value	PCB 126 log FC	PCB 126 Adj. *P*-value	Aroclor 1260 + PCB 126 logFC	Adj. *P*–value
Am (2-O-methyladenosine)	317398	0.0155	0.0203^*^	−0.1222	1.22e-14^*^	0.0965	5.61e-13^*^
m1A (1-methyladenosine)	27476	−0.1491	0.1465	−0.3532	0.0002^*^	0.0061	0.9630
m2,2,7G (N2,N2,7-trimethylguanosine)	341661811	−0.0355	0.8313	−0.2825	0.0058^*^	0.1379	0.3727
m2,2G (N2,N2-dimethylguanosine)	135501639	−0.0988	0.2639	−0.2403	0.0012^*^	0.0092	0.9630
m5U (5-methylurdine)	445408	−0.0735	0.7848	−0.2815	0.0056^*^	−0.0101	0.9630
m6A (N6-methyladenosine)	102175	−0.0679	2.99e-10^*^	−0.1246	0.0000^*^	0.0986	5.61e-13^*^
m6Am (N(6),O(2)-dimethyladenosine)	6453528	−0.0616	0.8313	−0.3065	0.0124^*^	0.1057	0.8059
m7G (7-methylguanosine)	135445750	−0.0384	0.8383	−0.5434	0.0013*	−0.0990	0.8843
ms2t6A 2-(methylsulfanyl)-N6-L-threonylcarbamoyladenine	254741220	−0.0116	0.8383	−0.1962	0.0012^*^	0.0933	0.1903
Ψ (pseudouridine)	15047	−0.2237	0.1465	−0.3785	0.0034^*^	0.0917	0.8059

^*^ Statistically significant as indicated by the Adj *P*-value in the Table.

**Table 2:** Enzymes that modify the RNA molecules identified in the HFD-fed mice exposed to PCBs and the roles of these modifications

RNA modification	RNA modified	Role	Writer	Eraser	Reader
Am	tRNA snRNAs	Structure and stability, RNA–RNA interaction [12]	FTSJ1 CMTR1 [41, 69, 70]	FTO [33]	
m1A	tRNA T-loop, mRNA, mt rRNA		TRMT6 TRMT61A TRMT61B (mt) [9, 71]	ALKBH1 [67], ALKBH3 [72]	YTHDF1–3 [10]
m2,2,7G	mRNA cap		TGS1 [39, 73]		
m2,2G	tRNA between the D-loop and anticodon loop		TRM1 (Trmt1) [74]		
m5U	tRNA		TRMT2A TRMT2B [43]		
m6A	mRNA and U6 RNA	Processing of pri-miRNA to pre-miRNA [75]; lncRNA, circRNAs, mRNA splicing, stability, translation, degradation [13, 57, 76–84]	METTL3 METTL14 WTAP VIRMA RBM15 RBM15B ZC3H13 WTAP CBLL1 METTL16 [55, 84–86]	FTO, ALKBH5 [55]	YTHDF1–3, YTHDC1–2, HNRNPA2B1, IGFBP1–3, PRRC2A, FMR1, ELAVL1, HNRNPC, HNRNPG [10, 79, 87–90]
m6Am	mRNA	Cap modification [52]	PCIF1 [91]	FTO [33]	
m7G	mRNA	Cap modification [52, 92, 93]	RNMT METTL1 BUD23 [53, 92]		
ms2t6A	tRNA	Position 37 of tRNAs responsible for codons starting with A (ANN)- stabilizes the anticodon loop structure and enhances tRNA-anticodon binding [94]	CDKAL1 [95]		
Ψ	tRNA, rRNA, snRNAs, mRNA	Stabilize tRNA structure [96], enhanced mRNA translation [97], role in mRNA not well-understood [98]	PUS1, PUS3, PUS7, PUS7L, PUS10, DKC1, TRUB1 [9, 10, 53]		Incorporation of Ψ suppressed RNA recognition by toll-like receptors TLR2, TLR2, and TLR8 [98]

mt = mitochondrial.

### PCB Exposure Affects RNA Modifications in HFD-fed Mouse Liver

Exposure of mice to Aroclor 1260 increased the severity of HFD-induced NAFLD [18] while generating the fewest alterations in the liver proteome [6]. Exposure of mice to HFD + Aroclor 1260 significantly altered the abundance of two RNA modifications, while HFD + PCB 126 altered 10 RNA modifications (Table 1). The abundance of all 10 RNA modifications was significantly reduced by PCB 126 exposure compared to HFD alone. In contrast, exposure to HFD + both Aroclor 1260 and PCB 126 resulted in significant alterations in only two of the 10 RNA modifications. Nine of the 10 RNA modifications detected were methylations. Methylation is the most abundant RNA chemical modification [12]. Only two modifications were significantly different from HFD in all three PCB exposure groups: 2ʹ-O-methyladenosine (Am), a tRNA and small nuclear RNA (snRNA) modification, and m6A, the most common modification of the mRNA, lncRNA, pri-miRNA transcriptome.

### RNA Modifications and the Expression of Their Writers, Readers and Erasers in HFD-fed Mouse Liver with PCB Exposure

The abundance of Am was increased in Aroclor 1260- and Aroclor 1260- + PCB 126-exposed mouse liver compared to HFD control, whereas exposure to PCB 126 reduced Am abundance (Table 1). 2ʹO-methylation of RNA affects the structure and stability including RNA–RNA hybrid interaction [12]. Neither the methyltransferase FTSJ1 (FtsJ RNA 2ʹ-O-Methyltransferase 1) nor the demethylase FTO (FTO Alpha-Ketoglutarate-Dependent Dioxygenase) that regulate Am modification (Table 2) were significantly altered in any PCB exposure group at the protein [6] or transcript level (Table 3).

**Table 3:** Expression of readers, writers and erasers of epitranscriptomic RNA marks in the HFD-fed PCB-exposed male mouse liver (All mice were on a HFD with four exposure groups: control, Aroclor 1260, PCB 126 or Aroclor 1260 + PDB 126 (Aro + PCB); data are the logFC relative to HFD control; all RNA transcript and protein values shown are significantly different in the direction indicated compared to HFD control (*P* < 0.05);. values are the mean of five separate mouse liver/exposure group.)

			RNA transcript abundance from RNA-seq				Protein abundance from (Jin *et al.* [16])		
RNA PTM	Writer	Ensembl (mouse gene ID)	Aroclor 1260 log FC	PCB 126 log FC	Aro + PCB logFC	Unitprot Protein ID	Aroclor 1260 log FC	PCB 126 log FC	Aro + PCB logFC
Am	FTSJ1	ENSMUSG00000031171				Q8CBC7			
	CMTR1	ENSMUSG00000024019				Q9DBC3			
m1A	TRMT6	ENSMUSG00000037376				Q8CE96			
	TRMT61A	ENSMUSG00000060950				Q80XC2		1.52	
	TRMT61B (mt)	ENSMUSG00000085492							
m2,2,7G	TGS1	ENSMUSG00000028233				Q923W1			
m2,2G	TRM1 (Trmt1)	ENSMUSG00000001909			−0.67	Q3TX08 A2RSY6			
m5U	TRMT2A	ENSMUSG00000022721			−0.53	Q8BNV1			
	TRMT2B	ENSMUSG00000067369				Q8BQJ6			
m6A	METTL3	ENSMUSG00000022160				Q8C3P			
m6A	METTL14	ENSMUSG00000028114				Q3UIK4			
m6A	WTAP	ENSMUSG00000060475				Q9ER69			
m6A	VIRMA	ENSMUSG00000040720				A2AIV2			
m6A	RBM15	ENSMUSG00000048109				Q0VBL3			
m6A	RBM15B	ENSMUSG00000074102				Q6PHZ5			
m6A	ZC3H13	ENSMUSG00000022000				E9Q784			
m6A	WTAP	ENSMUSG00000060475				Q9ER69			
m6A	CBLL1	ENSMUSG00000020659				Q9JIY2			
m6A	METTL16	ENSMUSG00000010554				Q9CQG2			
m6Am	PCIF1	ENSMUSG00000039849				Q6ZWS8			0.41
m7G	RNMT	ENSMUSG00000009535				Q9D0L8			
	METTL1	ENSMUSG00000006732				Q9Z120			
m7G	BUD23	ENSMUSG00000005378			−0.72	Q9CY21			
ms2t6A	CDKAL1	ENSMUSG00000006191				Q91WE6			
Ψ	PUS1	ENSMUSG00000029507			−0.65	Q9WU56		0.42	
	PUS3	ENSMUSG00000032103				Q9JI38			
	PUS7	ENSMUSG00000057541				Q91VU7			
	PUS7L	ENSMUSG00000033356				Q8CE46			
	PUS10	ENSMUSG00000020280			0.57	Q9D3U0			
	DKC1	ENSMUSG00000031403				Q9ESX5			
	TRUB1	ENSMUSG00000025086				Q8C0D0			
	Erasers								
Am, m6A, m6Am	FTO	ENSMUSG00000055932				Q8BGW1			
m1A, m6A	ALKBH1	ENSMUSG00000079036	−0.53			P0CB42			
m1A	ALKBH3	ENSMUSG00000040174				Q8K1E6			
m6A	ALKBH5	ENSMUSG00000042650				Q3TSG4			
	Readers								
m1A m6A	YTHDF1	ENSMUSG00000038848				P59326			
	YTHDF2	ENSMUSG00000040025				Q91YT7			
	YTHDF3	ENSMUSG00000047213			0.65	Q8BYK6			
m1A m6A	YTHDC1	ENSMUSG00000035851				E9Q5K9			
	YTHDC2	ENSMUSG00000034653			0.80	B2RR83			
m6A	HNRNPA2B1	ENSMUSG00000004980				O88569			
m6A	IGFBP1	ENSMUSG00000020429				P47876			
	IGFBP2	ENSMUSG00000039323	−0.62		−0.98	P47877			
	IGFBP3	ENSMUSG00000020427				P47878			
m6A	PRRC2A	ENSMUSG00000024393			−0.73	Q7TSC1			
	FMR1	ENSMUSG00000000838	0.67		0.87	P35922	0.67		0.66
	ELAVL1	ENSMUSG00000040028				P70372			
	HNRNPC	ENSMUSG00000060373				Q5RA82			
	HNRNPG = Rbmx	ENSMUSG00000031134				Q9WV02			

Am is converted to N^6^,2ʹ-O-dimethyladenosine (m6Am) [33]. The enzyme Phosphorylated CTD Interacting Factor 1 (PCIF1) catalyzes m6A methylation on 2-O-methylated adenine located at the 5′ ends of mRNAs to yield m6Am [34]. m6Am is in mRNA, rRNA, tRNA, non-coding RNA [13] and mitochondrial rRNA [35]. m6Am abundance was reduced in the PCB 126 exposure samples compared to HFD control. m6Am is the first nucleotide after the 7-methylguanosine (m7G) cap of selected mRNAs [36]. Increased m6Am corresponds with increased PCIF1 writer protein expression in HFD + PCB 126- + Aroclor 1260-exposed mouse liver compared to HFD control (Table 2). PCIF1 protein was not altered in the other PCB-exposed mice.

The abundance of m7G, a cap for mRNA [37], was significantly reduced by PCB 126 exposure (Table 1). Likewise, a decrease in BUD23 RRNA Methyltransferase and Ribosome Maturation Factor (Bud23) transcript abundance was detected in Aroclor 1260 + PCB 126 compared to HFD alone (Table 3). BUD23 methylates m7G in 18S rRNA [38]. N2,N2,7-trimethylguanosine (m2,2,7 G) abundance was also significantly reduced by PCB 126 exposure. m2,2,7G is an mRNA cap [39]. The transcript levels of Trmt1 (tRNA (m2,2,G2,6) dimethyltransferase) were significantly reduced in the mice exposed to Aroclor 1260 and PCB 126 compared to HFD control (Supplementary Table S2) but were not significantly changed by exposure to PCB 126 alone. Cap Methyltransferase 1 (CMTR1) methylates the ribose of the first nucleotide of an m(7)GpppG-capped mRNA [40] and other ribonucleosides, e.g. Am in mRNA [41]. We did not detect any significant change in Cmtr1 transcript or CMTR1 protein expression in the liver of any of the exposure groups (Table 3).

2-Methylthio-N^6^-threonylcarbamoyladenosine (ms2t6A) is in tRNA and is essential for decoding of Lys codons [42]. ms2t6A abundance was significantly reduced by PCB 126 exposure in the HFD mouse livers (Table 1). There was no significant alteration of its methyltransferase CDK5 Regulatory Subunit Associated Protein 1 Like 1 (CDKAL1) in the transcriptome or proteome analysis of the HFD ± PCB exposure mouse livers.

Ψ is present in the tRNA anticodon loop and is an internal mRNA modification [9, 10]. Ψ abundance was significantly reduced by PCB 126 exposure in the HFD mouse livers (Table 1). A number of enzymes are involved in the conversion of U to Ψ (Table 2). Pseudouridine Synthase 1 (PUS1) protein was increased in the livers of the HFD + PCB 126-exposed mice (Table 3). We detected a reduction in Pus1 and an increase in Pseudouridine Synthase 10 (Pus10) transcript levels in HFD-fed mice exposed to both Aroclor 1260 and PCB 126 (Table 3). m1A abundance was significantly reduced by PCB 126 exposure in the HFD mouse livers (Table 1). Like m6A, m1A is a reversible mark added by three writers, erased by AlkB Homolog 1 and 3 (ALKBH1 and ALKBH3, Histone H2A Dioxygenases) and read by readers YTH N6-Methyladenosine RNA Binding Proteins 1–3 (YTHDF1–3; Table 2). Despite the reduced m1A abundance, we detected a significant increase in TRMT61A (mRNA Methyladenosine-N(1)-Methyltransferase Catalytic Subunit TRMT61A) m1A writer protein in the livers of the HFD + PCB 126-exposed mice. We detected a significant reduction in Alkbh1 transcript levels in the livers of HFD mice exposed to Aroclor 1260 (Table 3). We detected significant increases in the transcript abundance of the m1A readers Ythdf3 and Ythdc2 in the livers of HFD mice exposed to both Aroclor 1260 and PCB 126 (Table 3). Taken together, none of the transcript or protein expression changes in the m1A writers, readers or erasers correlate with the observed decrease in m1A abundance in the livers of the HFD mice exposed to PCB 126.

Exposure of mice to HFD and either Aroclor 1260 or PCB 126 individually or in combination also reduced the abundance of 5-methyluridine (m5U), a modification found in the T-loop of tRNA [9] and catalyzed by TRMT2A and TRMT2B in mammals [43]. Interestingly, we detected a significant reduction in Trmt2a transcript levels in the HFD-fed mice exposed to both Aroclor 1260 and PCB 126; however, there was no significant difference in the protein levels of TRMT2A or TRMT2B in the proteome from these mice [6] (Table 3).

### Integrated Network Analysis of RNA Modifications Identified in Mouse Liver after HFD and PCB Exposures

**Figure 2:**
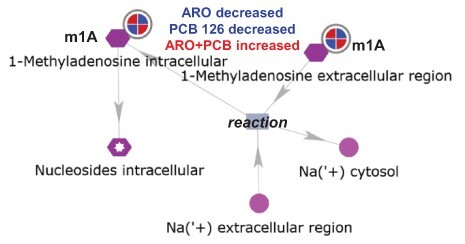
m1A network in PCB- and HFD-exposed mouse liver: the RNA modifications detected in HFD mouse liver after exposure to (i) Aroclor 1260, (ii) PCB 126 or (iii) Aroclor 1260 + PCB 126, with log FC and *P* values were analyzed together using network analysis filtered by tissue (liver) and species (mouse) in MetaCore. The circle with red and blue wedges depicts the significant reduction (blue) of m1A in Aroclor 1260- and PCB 126-exposed mice on HFD and the increase (red) in m1A observed in the Aroclor 1260- + PCB-exposed mice on HFD. MetaCore connected extracellular m1A to an intracellular reaction that regulates Na+ in the cytosol, but we were unable to ascertain information on this connection in PubMed

**Figure 3:**
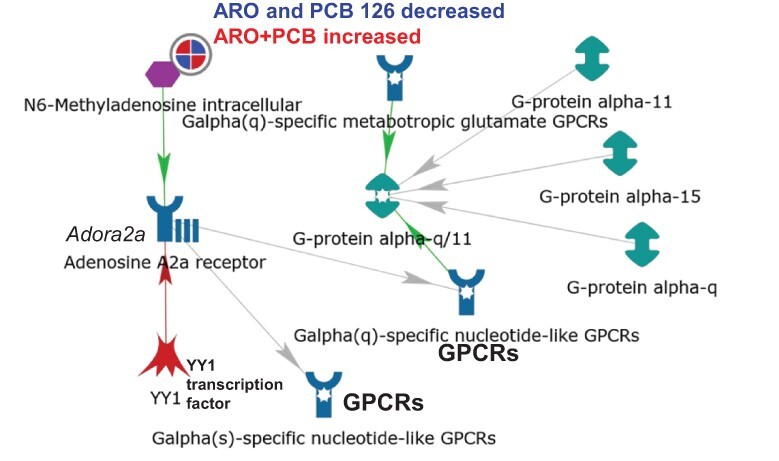
m6A network in PCB- and HFD-exposed mouse liver connects through activation of adenosine A2a receptor (*Adora2a*) to G protein-coupled receptor (GPCR) signaling pathways. The RNA modifications detected in HFD mouse liver after exposure to (i) Aroclor 1260, (ii) PCB 126 or (iii) Aroclor 1260 + PCB 126, with log FC and *P* values, were analyzed together using network analysis filtered by tissue (liver) and species (mouse) in MetaCore. The circle with red and blue wedges depicts the significant reduction (blue) of m6A in Aroclor 1260- or PCB 126-exposed mice and the increase (red) in m6A observed in the livers of mice with combined Aroclor 1260 + PCB 126 exposure. Green lines and arrows indicate positive/activation, whereas red indicates negative/inhibition. Whether YY1 inhibits *Adora2a* transcription in mouse liver is as depicted was not found in PubMed

Integrated network analysis of the 10 significant RNA modifications (Table 1) in MetaCore filtered by tissue (liver) and species (mouse) identified two networks in all three HFD mouse liver PCB exposure groups: (i) ‘m1A intracellular’ (Fig. 2) and (ii) ‘m6A intracellular’ (Fig. 3). A limitation of MetaCore is that it relies on published information and there are very few publications on the PTM of mouse liver RNA. We examined the expression of the identified upstream or downstream network proteins/genes in the proteome and RNA-seq transcriptome from each mouse liver exposure group, but no differences in expression versus the HFD control were detected (Supplementary Table S3).

### Identification of Joint Pathways for RNA Modifications and the Transcriptome Identified in Mouse Liver after HFD and PCB Exposures

MetaCore was used to integrate the RNA modification and transcriptome data of the mouse liver in response to HFD and exposure to each PCB (Tables 4 and 5). This analysis revealed pathways driven by the more numerous changes in the RNA transcriptome in each HFD mouse liver PCB exposure sample than the RNA modifications alone. This result likely reflects the paucity of literature on how RNA modifications impact the transcriptome in mouse liver.

**Table 4:** MetaCore network analysis and GO processes from comparative analysis of RNA modification in three PCB exposure mouse liver data sets (all versus HFD control): HFD + Aroclor 1260; HFD + PCB 126; HFD + Aroclor 1260 + PCB 126

Network	GO processes
m1A intracellular	Receptor metabolic process (100.0%; 1.184e-15) Receptor-mediated endocytosis (100.0%; 1.961e-13) Low-density lipoprotein particle receptor catabolic process (57.1%; 3.692e-12) Low-density lipoprotein receptor particle metabolic process (57.1%; 3.692e-12) Cell surface receptor signaling pathway involved in cell–cell signaling (100.0%; 9.348e-12)
m6A intracellular	Phosphatidylinositol-mediated signaling (40.0%; 7.977e-21) Inositol lipid-mediated signaling (40.0%; 1.359e-20) Response to organonitrogen compound (73.3%; 5.222e-18) Response to nitrogen compound (73.3%; 2.047e-17) Phospholipase C-activating G protein-coupled receptor signaling pathway (36.7%; 6.330e-17)

**Table 5:** MetaCore pathway maps for each mouse liver exposure group

Exposure (all HFD)	Pathway map 1	Pathway map 2	Pathway map 3	Pathway map 4
Aroclor 1260, PCB 126 or Aroclor 1260 + PCB 126	Immune response: role of DPP4 (CD26) in immune regulation	Cytidine triphosphosphate (CTP)/uridine triphosphosphate (UTP) metabolism	Role of interferon (IFN)-beta in the improvement of blood–brain barrier integrity in multiple sclerosis	Regulatory T cells in murine model of contact hypersensitivity

## Discussion

PCBs are ‘legacy contaminants’ that act as metabolism-disrupting chemicals and that continue to be present in humans (reviewed in [44]), including increased levels in circulation after weight reduction surgery in obese individuals [1, 45]. In mouse models on a HFD, NDL PCBs, e.g. Aroclor 1260, increased diet-induced hepatic steatosis, inflammation and fibrosis by affecting pathways regulated by epidermal growth factor receptor (46–49), constitutive androstane receptor (CAR, *Nr1i3*) and Pregnane X receptor (PXR, *Nr1i2*) [3, 16, 17]. In contrast, exposure of mice to a ‘healthy diet’ and DL PCBs, e.g. PCB-126, but not NDL PCBs, e.g. Aroclor 1260, results in hepatic steatosis and NAFLD [2]. We previously reported large differences in the liver proteome of mice exposed to Aroclor 1260, PCB 126 and the combination of Aroclor 1260 + PCB 126 and identified many new PCB targets that explain in part some of the differences in liver phenotypes with these PCBs [6]. The rationale for our study is based on the observation that exposure to Aroclor 1260 + PCB 126 was associated with GO processes related to the regulation of gene expression by epigenetic mechanisms [6]. In addition, epitranscriptomic RNA modifications alter the processing of primary RNA transcripts and mRNA translation. Thus, here, we examined the global epitranscriptome in all three PCB exposure groups to address the idea that epitranscriptomic reprogramming regulates the proteome and mRNA transcriptome. A weakness in the present study is the absence of the evaluation of control diet versus HFD that can be addressed in future work.

To our knowledge, this is the first study to identify global RNA epitranscriptomic changes by MS in the livers of mice exposed to HFD and PCBs that cause hepatic steatosis and fibrosis. Examination of RNA chemical modification in response to the environmental exposure and the impact of these epitranscriptomic marks on environmental disease is a growing field of interest [14]. To date, most studies have examined the impact of selected toxicants, e.g. B[a]P, aflatoxin B1, BPA, pesticides and arsenic, on the transcript levels of m6A readers, writers and erasers in mouse tissues or cell lines [14]. The state of knowledge on the contribution of m6A epitranscriptomics to hepatic function and liver disease was recently reviewed [13], thus identifying significant gaps in knowledge about this well-studied modification. However, other modifications are still barely examined. Exposure of zebrafish embryos to 10 nM PCB 126 for 7 h followed by m6A-RIP-seq identified 15 differential m6A peaks in transcripts of PCB-exposed embryos [50]. The 15 m6A-marked transcripts after PCB 126 exposure are targets of AHR agonists (fitting with this DL PCB) and the study identified one differential splicing event, indicating that PCB 126 can affect developmental gene expression patterns [50]. m6A RIP in HepaRG cells identified *UGT2B7* as downregulated by m6A modification [51]. There are species and tissue/cell-type differences in m6A marks in the epitranscriptome of mice and humans [52].

Our results support the possible role of m6A modification in HFD and PCB exposure-induced mouse liver steatosis. m6A abundance was reduced in total hepatic RNA from mice on HFD with exposure to Aroclor 1260 or PCB 126; however, when mice were exposed to the combination of Aroclor 1260 and PCB 126, m6A abundance was increased compared to HFD control. This finding may relate to the increase in GO ‘Regulation of Gene Expression Processes’ in the liver proteome of the HFD + co-exposed Aroclor 1260 + PCB 126 mice [6].

There are a few studies of m6A in mouse liver. The expression of m6A readers, writers and erasers was reduced from birth to 60 days in the livers of male C57Bl/6J mice [53]. HFD-fed female C57BL/6 mice (6 weeks, 45% fat) had decreased liver m6A levels and increased FTO protein expression [54]. FTO is an ‘eraser’ of the m6A mark [55]. Mice with global knockout of Mettl3 (an m6A writer) showed a decreased expression of genes regulating liver fatty acid (FA) synthesis, e.g. *Pgc1a1*, *Fasn* and *Sirt1* ([56], reviewed in [13]). HFD-fed (8 weeks 45% fat) male C57Bl/6 mice had 6254 more m6A peaks identified in m6A-RNA immunoprecipitation-sequencing (Me-RIP-seq) in the liver compared to control diet–fed mice [57]. The authors reported increased m6A peaks in genes associated with lipid metabolism–associated GO processes, i.e. peroxisome proliferator-activated receptor (PPAR) signaling pathway, FA degradation, FA metabolism and biosynthesis of unsaturated FA in the HFD liver. In contrast, ‘downmethylated’ genes in the HFD liver were enriched in GO terms ribosome, steroid hormone biosynthesis, chemical carcinogenesis, linoleic acid metabolism and retinol metabolism [57].

Another study showed that glucocorticoid-induced lipid accumulation in liver cells was mediated by glucocorticoid receptor–induced FTO expression resulting in decreased m6A and activation of lipogenic genes, e.g. *Scd* [58]. Similarly, Me-RIP-seq identified more m6A marks in liver transcripts from obese *db/db* male mice compared to lean mice with increased m6A abundance and transcript expression of lipogenic genes, e.g. *Srebp1c*, *Fasn*, *Acc1* and *Scd* [59]. Despite these studies demonstrating that HFD affects liver m6A and FTO, there are no studies examining global epitranscriptomic RNA modifications in the liver after environmental exposure in the presence of HFD, thus making this study unique.

Me-RIP-seq identified 6254 more m6A peaks in the liver from HFD-fed male C57Bl/6 mice compared to control diet–fed mice [57]. Thus, the finding of reduced total m6A in the HFD male Aroclor 1260- or PCB 126-exposed mice in our study suggests that exposure to PCBs may reduce m6A levels, while the combination of both Aroclor 1260 and PCB 126 mitigated this response. It will be important to identify transcripts modified by m6A in our mouse model of TASH by Me-RIP-seq for a direct comparison of hepatic responses to these environmental stressors.

We observed that m7G abundance was reduced by PCB 126 exposure along with Bud23, a m7G methyltransferase, transcript expression. Bud23 (previously called Wbscr22) was significantly reduced from birth to 60 days in the livers of male C57Bl/6J mice [53]. BUD23 and TRMT112 form a heterodimeric methyltransferase nucleocytoplasmic complex for rRNA m7G modification [38]. This is an essential step in the 40S biogenesis pathway required for protein translation [60]. mRNAs are recruited to the ribosome by m7G interaction with the cap-binding complex eIF4E, eIF4A and eIF4G [61]. Thus, we would expect a reduction in protein synthesis; indeed, Aroclor 1260 + PCB 126 exposure generated 14 fewer proteins in the hepatic proteome analysis compared to the PCB 126 proteome but 109 more proteins compared to the Aroclor 1260 proteome [6]. However, eIF4E3 protein was increased in the PCB 126 proteome (1.44 logFC) compared to HFD and was not significantly altered in the other PCB exposure proteomes [6]. Since eIF4E3 inhibits mRNA export and translation targets of eIF4E1 and acts as a tumor suppressor [62], the increase in eIF4E3 in the PCB 126 proteome may affect the selected transcript translation.

Similarly, mRNA cap m2,2,7G abundance was reduced by PCB 126 exposure. m2,2,7G is also a cap for uridine-rich snRNAs that are recognized by SNUPN (Snurportin 1) [63]. There was no significant difference in liver Snupn transcript or protein abundance between HFD control and HFD + PCB 126-exposed mice (data not shown).

Ψ abundance was significantly reduced by PCB 126 exposure in the HFD mouse livers. Despite the high levels of Ψ in RNA, there is only one study on the impact of environmental exposures on Ψ in mouse liver [64]. That study showed an increase in Ψ in the urine of mice after a dietary exposure to a mixture of six pesticides: boscalid, captan, chlorpyrifos, thiofanate, thiacloprid and ziram [64]. Similarly, exposure of mice to Ochratoxin A (a mycotoxin) increased Ψ in urine and was considered a marker of renal cell turnover [65]. m1A abundance was significantly reduced by PCB 126 exposure in the HFD mouse livers. The number of m1A modifications in the mRNA transcriptome varies with the detection method [10] but is generally found in the 5ʹUTR of some mRNAs [66]. m1A is also found in the T-loop of tRNA [9]. Decreased m1A abundance in tRNA affects the translation initiation and elongation by regulating the levels of initiator methionyl-tRNA (RNA^iMet^) and the association of other ALKBH1-target tRNAs to polysomes, thus affecting protein synthesis [67].

## Conclusion

This study identified global changes in the PTM RNA transcriptome in a murine model of PCB and HFD-exposure-induced TASH. Environmental hepatology is an emerging field focused on identifying and treating environmental contributions to liver disease [68]. From 22 PTM global RNA modifications detected, we identified 10 RNA modifications that were significantly changed by PCB exposure in HFD-fed male mice. The abundance of m6Am was associated with the highest number of hepatic transcript changes in HFD + PCB 126- + Aroclor 1260-exposed mice. Increases in the abundance of m6Am and Ψ were associated with increased PCIF1 and PUS10 writer protein expression in HFD + PCB 126- + Aroclor 1260-exposed mouse liver. Increased m1A and m6A were associated with increased reader YTHDF2 and YTHDC2 transcript and reader FMR1 transcript and protein abundance. Further studies identifying transcripts directly regulated by these epitranscriptomic RNA modifications are needed to contextualize their role in TASH and evaluate if similar modifications occur in human disease.

## Supplementary Material

dvab008_Supp

## Data Availability

Data are available in Supplementary data.
