# Supplementary material for: Combined exposure to polychlorinated biphenyls and high-fat diet modifies the global epitranscriptomic landscape in mouse liver
Source: Environ Epigenet. 2021 Sep 17;7(1):dvab008. doi: 10.1093/eep/dvab008 (PMC8448424; doi:10.1093/eep/dvab008)
Supplement: dvab008_Supp [file dvab008_supp.zip › SupplementaryTables and Sup Fig 1REVISED.pdf]

Supplementary Table 1: RNA modifications detected in mouse liver. Values are the mean log fold change (logFC) from five independent mice/exposure group. Where logFC is the logarithm of the fold change with base 2 and the adjusted (Adj.) p value is the adjustment for the raw p-value using BH method for multiple testing of chemicals (Benjamini and Hochberg 1995).

			Arochlor1260 vs HFD		PCB 126 vs HFD		Arochlor1260 + PCB 126 vs HFD	
**Chemical name**	**PubChem CID**	**modificatior**	**logFC**	**dj.p-valu**	**logFC**	**dj.p-valu**	**logFC**	**adj.p-value**
2-O-methyladenosine	317398	Am	0.0155	**0.0203**	-0.1222	**0.0000**	0.0965	**0.0000**
2'-O-methylcytidine	145710370	Cm	-0.0305	0.8313	-0.0966	0.2700	0.0740	0.8059
2'-O-methylguanosine	135406950	Gm	0.4654	0.7848	0.3206	0.5901	0.1194	0.9630
1-methyladenosine	27476	m1A	-0.1491	0.1465	-0.3532	**0.0002**	0.0061	0.9630
1-methylguanosine	96373	m1G	-0.2016	0.1465	-0.1892	0.0704	0.0169	0.9630
N2,N2,7-trimethylguanosine	341661811	m2,2,7G	-0.0355	0.8313	-0.2825	**0.0058**	0.1379	0.3727
N2,N2-dimethylguanosine	135501639	m2,2G	-0.0988	0.2639	-0.2403	**0.0012**	0.0092	0.9630
N2-methylguanosine	135501934	m2G	-0.5758	0.5199	-0.9535	0.0758	-0.4250	0.8059
3-methyluridine	316991	m3U	0.0243	0.9009	-0.3038	0.1906	0.0563	0.9630
N4-methylcytidine	101436	m4C	-0.1199	0.8313	-0.2604	0.2217	-0.0786	0.9630
5-methylcytidine	92918	m5C	-0.0751	0.4105	-0.1034	0.0762	0.1109	0.1903
5-Methyluridine	445408	m5U	-0.0735	0.7848	-0.2815	**0.0056**	-0.0101	0.9630
5,2'-O-Dimethyluridine	14367009	m5Um	-0.1442	0.8313	-0.0431	0.8638	0.0003	0.9990
N6,N6-Dimethyladenosine	440004	m6.6A	0.0661	0.8313	-0.3080	0.0758	0.1032	0.9206
N6-Methyladenosine	102175	m6A	-0.0679	**0.0000**	-0.1246	**0.0000**	0.0986	**0.0000**
N(6),O(2)-Dimethyladenosine	6453528	m6Am	-0.0616	0.8313	-0.3065	**0.0124**	0.1057	0.8059
7-methylguanosine	135445750	m7G	-0.0384	0.8383	-0.5434	**0.0013**	-0.0990	0.8843
2-(Methylsulfanyl)-N6-L-threonylcarbamoyladenine	254741220	ms2t6A	-0.0116	0.8383	-0.1962	**0.0012**	0.0933	0.1903
N6-Succinyl AdenosineR	165243	N6-SA	-0.3450	0.8313	-0.1061	0.8638	-0.1331	0.9630
N6-threonylcarbamoyladenosine	161466	t6A	-0.0540	0.8383	-0.2255	0.3473	0.0357	0.9630
2'-O-methyluridine	102212	Um	-0.0651	0.8313	-0.1598	0.3303	0.0294	0.9630
Pseudouridine	15047	Y	-0.2237	0.1465	-0.3785	**0.0034**	0.0917	0.8059

Supplementary Table 2: Comparison of transcript abundance for writers, readers, and erasers of RNA PTM in RNA seq reported here and protein expression from proteome analysis (Jin et al. 2020). P is p value.

RNA PTM	target	Writer	Ensembl (mouse gene ID)	Aroclor 1260 log FC	P	PCB 126 log FC	P	Aro + PCB logFC	P	Protein ID	Aroclor 1260 log FC	P	PCB 126 log FC	P	Aro + PCB logFC	P
Am	tRNA	FTSJ1	ENSMUSG00000031171							Q8CBC7						
	snRNAs	CMTR1	ENSMUSG00000024019							Q9DBC3						
m1A	tRNA T-loop, mRNA	TRMT6	ENSMUSG00000037376							Q8CE96						
		TRMT61A	ENSMUSG00000060950							Q80XC2			1.52	6.09E-03		
		TRMT61B (mt)	ENSMUSG00000085492													
m2,2,7G	mRNA cap	TGS1	ENSMUSG00000028233							Q923W1						
m2,2G	tRNA between the D-loop and anticodon loop	TRM1 (Trmt1)	ENSMUSG00000001909					-0.67	5.94E-08	Q3TX08 A2RSY6						
m5U	tRNA	TRMT2A	ENSMUSG00000022721					-0.53	6.12E-05	Q8BNV1						
		TRMT2B	ENSMUSG00000067369							Q8BQJ6						
m6A	mRNA and U6 RNA	METTL3	ENSMUSG00000022160							Q8C3P						
		METTL14	ENSMUSG00000028114							Q3UIK4						
		WTAP	ENSMUSG00000060475							Q9ER69						
		VIRMA	ENSMUSG00000040720							A2AIV2						
		RBM15	ENSMUSG00000048109							Q0VBL3						
		RBM15B	ENSMUSG00000074102							Q6PHZ5						
		ZC3H13	ENSMUSG00000022000							E9Q784						
		WTAP	ENSMUSG00000060475							Q9ER69						
		CBLL1	ENSMUSG00000020659							Q9JIY2						
		METTL16	ENSMUSG00000010554							Q9CQG2						
m6Am	mRNA cap modification	PCIF1	ENSMUSG00000039849							Q6ZWS8					0.41	3.01E-02
m7G	mRNA cap modification	RNMT	ENSMUSG00000009535							Q9D0L8						
		METTL1	ENSMUSG00000006732							Q9Z120						
		BUD23	ENSMUSG00000005378					-0.72	7.52E-08	Q9CY21						
ms2t6A	tRNA	CDKAL1	ENSMUSG00000006191							Q91WE6						
Ψ	tRNA-anticodon loop, snRNAs	PUS1	ENSMUSG00000029507					-0.65	2.96E-05	Q9WU56			0.42	8.39E-03		
		PUS3	ENSMUSG00000032103							Q9JI38						
		PUS7	ENSMUSG00000057541							Q91VU7						
		PUS7L	ENSMUSG00000033356							Q8CE46						
		PUS10	ENSMUSG00000020280					0.57	3.19E-05	Q9D3U0						
		DKC1	ENSMUSG00000031403							Q9ESX5						
		TRUB1	ENSMUSG00000025086							Q8C0D0						
Am, m6A, m6Am	Erasers	FTO	ENSMUSG00000055932							Q8BGW1						
m1A, m6A		ALKBH1	ENSMUSG00000079036	-0.53	1.59E-03					P0CB42						
m1A		ALKBH3	ENSMUSG00000040174							Q8K1E6						
m6A		ALKBH5	ENSMUSG00000042650							Q3TSG4						
m1A m6A	Readers	YTHDF1	ENSMUSG00000038848							P59326						
		YTHDF2	ENSMUSG00000040025							Q91YT7						
		YTHDF3	ENSMUSG00000047213					0.65	1.40E-05	Q8BYK6						
m1A m6A		YTHDC1	ENSMUSG00000035851							E9Q5K9						
		YTHDC2	ENSMUSG00000034653					0.80	1.93E-04	B2RR83						
m6A		HNRNPA2B1	ENSMUSG00000004980							O88569						
		IGFBP1	ENSMUSG00000020429							P47876						
		IGFBP2	ENSMUSG00000039323	-0.62	6.71E-03			-0.98	1.72E-05	P47877						
		IGFBP3	ENSMUSG00000020427							P47878						
		PRRC2A	ENSMUSG00000024393					-0.73	3.55E-06	Q7TSC1						
		FMR1	ENSMUSG00000000838	0.67	5.57E-04			0.87	7.48E-06	P35922	0.67	1.07E-03			0.66	8.56E-04
		ELAVL1	ENSMUSG00000040028							P70372						
		HNRNPC	ENSMUSG00000060373							Q5RA82						
		HNRNPG = Rbmx	ENSMUSG00000031134							Q9WV02						

Supplementary Table 3: MetaCore Network analysis of genes in m1A and m6A networks with the expression of RNA transcripts from RNA seq presented here and the proteins identified in the proteome analysis of liver from the HFD-fed mice exposed to Aroclor 1260, PCB 126, or the combination of Aroclor 1260 + PCB 126.			Expression in proteome (Jin et al. 2020)		
**m1A network**	**mouse gene**	**UniProt**	**Aroclor 1260 FC**	**PCB 126 FC**	**Aro + PCB FC**
G protein-activated inward rectifier potassium channel 1 (GIRK)	*Kcnj3*	P63250	ND	ND	ND
Serine/threonine-protein kinase mTOR	*Mtor*	Q9JLN9	ND	ND	ND
**m6A network**					
Adenosine A2a receptor	*Adora2a*	Q60613	ND	ND	ND
G protein alpha 15	*Gna15*	P30678	ND	ND	ND
Transcriptional repressor protein YY1	*Yy1*	Q00899	ND	ND	ND
Phosphatidylinositol 3-kinase regulatory subunit alpha (p85)	*Pik3r1*	P26450	ND	ND	ND

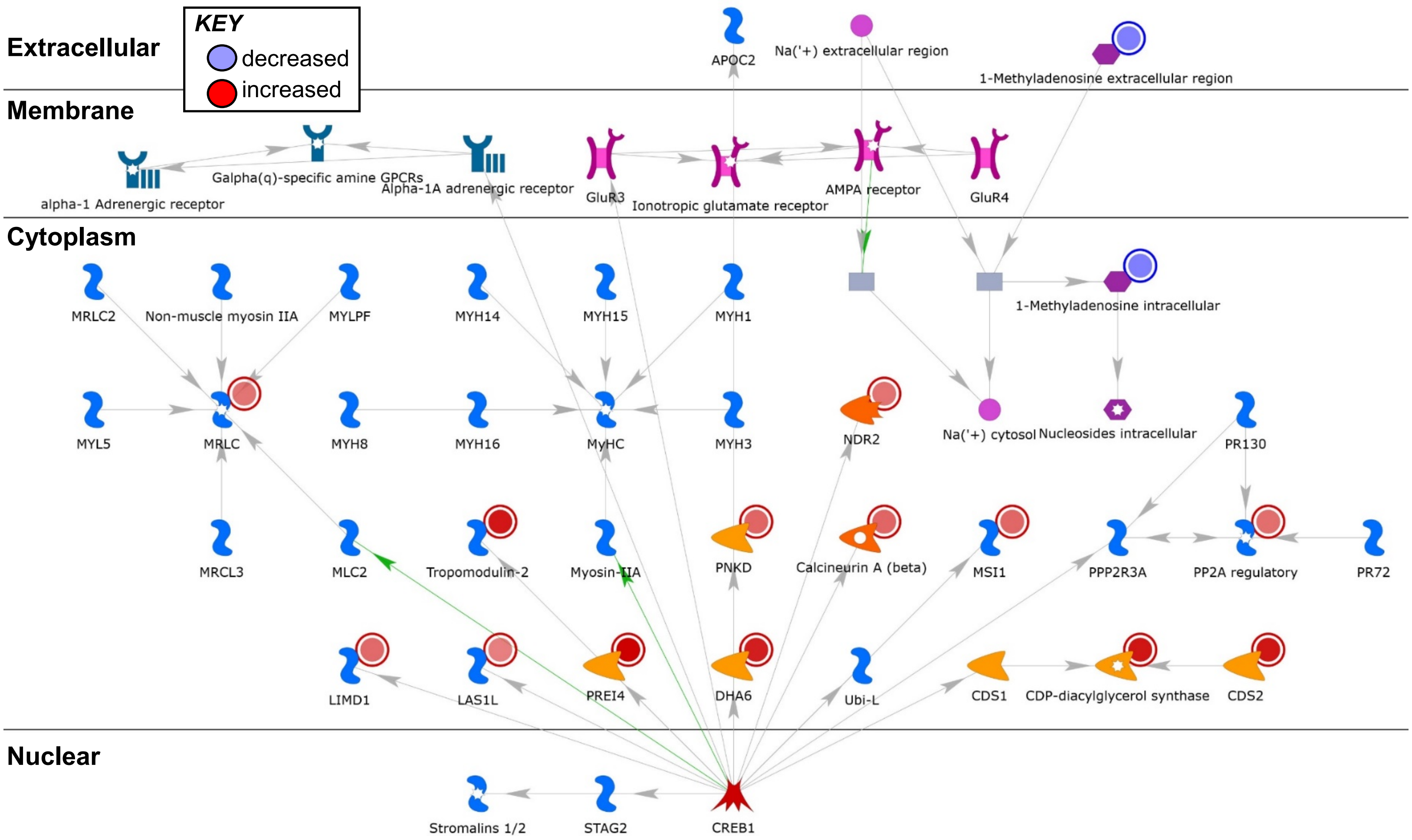

Supplementary Figure 1: Network #6 identified in joint analysis of RNA modifications and proteome in HFD-fed mice exposed to PCB 126 versus HFD control: 1mA extracellular region, 1mA intracellular, LAS1L, MSI1, LIMD1. From the RNA modification data we see the reduction (blue circles) in m1A abundance (extracellular and intracellular). From the proteome data we see increased (red circle): MRLC (Myl7) is myosin light chain 7; NDR2 (Stk38l) is a ser/thr protein kinase; Tropo modulin-2 (Tmod2) is an actin-regulating protein, PNKD (PNKD Metallo-Beta-Lactamase Domain Containing) regulates myofibrillogenesis; Calcineurin A (beta) (Ppp3cb) is the catalytic subunit of Ser/Thr-Protein Phosphatase 2B that is a calcium-dependent, calmodulin-stimulated protein phosphatase trat transduces intracellular Ca++ signals; MSI1 (musashi RNA binding protein 1) is an RNA binding protein that regulates the expression of target mRNAs at the translation level; PP2A regulatory (Ppp2R5d) is a component of the ser/thr Protein phosphatase 2A complex; LIMD1 (LIM domains containing 1) is a scaffold protein that positively regulates miRNA-mediated gene silencing and is essential for P-body formation; LAS1L (LAS1 like ribosome biogenesis factor) is involved in the biogenesis of the 60S ribosomal subunit and is required for maturation of the 28S rRNA; PREI4 (Gpcpd1) is Glycerophosphocholine phosphodiesterase 1; DHA6 (Aldh1a3) is Aldehyde dehydrogenase 1 family member A3; CDP-diacylglycerol synthase (Cds1) catalyzes the conversion of phosphatidic acid to CDP-diacylglycerol; CDS2 (CDP-diacylglycerol synthase 2).
